# Oxidative Stress as a Pathophysiological Core of Obesity: Preclinical Evidence for Antioxidant-Based Therapy Strategies

**DOI:** 10.3390/ph19091414

**Published:** 2026-09-07

**Authors:** Mariana Maciel Pereira, Carolinne Souza de Amorim, Aline Cristina Casimiro de Albuquerque Gomes, Helber da Maia Valenca, Mariana Renovato-Martins, Manuella Lanzetti, Samuel Santos Valenca, João Alfredo de Moraes

**Affiliations:** 1 Programa de Pós-Graduação em Farmacologia e Química Medicinal, Instituto de Ciências Biomédicas, Universidade Federal do Rio de Janeiro, Rio de Janeiro 21941-902, RJ, Brazil; macielmariana.nutri@gmail.com; 2 Instituto de Ciências Biomédicas, Universidade Federal do Rio de Janeiro, Rio de Janeiro 21941-902, RJ, Brazil; carolinne28amorim@hotmail.com (C.S.d.A.); aline_casimiro@hotmail.com (A.C.C.d.A.G.); manuellalanzetti@icb.ufrj.br (M.L.); joaomoraes@icb.ufrj.br (J.A.d.M.); 3 Programa de Pós-Graduação em Ciências Morfológicas, Instituto de Ciências Biomédicas, Universidade Federal do Rio de Janeiro, Rio de Janeiro 21941-902, RJ, Brazil; helfarma@gmail.com; 4 Departamento de Biologia Celular e Molecular, Instituto de Biologia, Universidade Federal Fluminense, Niterói 24210-201, RJ, Brazil; marianarenovato@id.uff.br

**Keywords:** oxidative stress, antioxidant therapy, adipose tissue dysfunction, Nrf2/AMPK signaling, preclinical obesity models

## Abstract

Obesity is increasingly recognized as a complex metabolic disorder characterized by persistent redox imbalance, rather than merely excess body weight. Its pathophysiology extends beyond energy imbalance to encompass chronic redox disruption. Expansion of adipose tissue, particularly in visceral depots, exceeds mitochondrial capacity, impairs antioxidant defenses such as superoxide dismutase, catalase, and glutathione peroxidase, and perpetuates chronic low-grade inflammation via nuclear factor kappa B (NF-kB) and nicotinamide adenine dinucleotide phosphate (NADPH) oxidase pathways. This review synthesizes preclinical evidence for diverse interventions, including vitamins A, B, C, E, and D; minerals such as zinc and selenium; amino acids such as N-acetylcysteine (NAC), L-carnitine, and taurine; various antioxidant compounds; and approved drugs including metformin, exenatide, fenofibrate, and orlistat. Despite differing structures and mechanisms, these interventions converge on restoring redox balance by activating nuclear factor erythroid 2–related factor 2 (Nrf2)/AMP-activated protein kinase (AMPK), increasing glutathione, and stabilizing mitochondria. However, translation of these preclinical findings into clinical practice requires further clarification of dosing, delivery methods, and long-term safety.

## 1. Introduction

Obesity represents a significant health challenge in the 21st century. It is not merely a matter of appearance or lifestyle. Rather, obesity is a chronic disease with multifactorial origins, affecting hundreds of millions globally [1]. Its primary characteristic is excessive or abnormal fat accumulation, which can impair health and contribute to metabolic, cardiovascular, and oncological conditions [2]. The World Health Organization (WHO) defines obesity using the Body Mass Index (BMI), calculated as weight in kilograms divided by height in meters squared. Adults with a BMI of 25 kg/m^2^ or higher are classified as overweight, while a BMI greater than 30 kg/m^2^ indicates obesity [3]. Although BMI is widely used in clinical practice, it does not differentiate between lean body mass (muscle, bone, organs, and water) and fat mass, nor does it account for the distribution of adiposity, thereby limiting its capacity to specify individual health risks [4].

A significant advance was proposed by The Lancet Diabetes & Endocrinology Commission in January 2025, which recommended conceptualizing obesity as a disease with distinct clinical phenotypes rather than solely by BMI. The Commission distinguished preclinical obesity, characterized by excess adiposity without organ or metabolic dysfunction, from clinical obesity, defined by organ dysfunction, metabolic disturbances, or functional impairment directly attributable to adiposity. This two-tiered framework links underlying disease mechanisms with patient manifestations, providing a more precise approach to diagnosis and treatment guidance [1].

**Figure 1:**
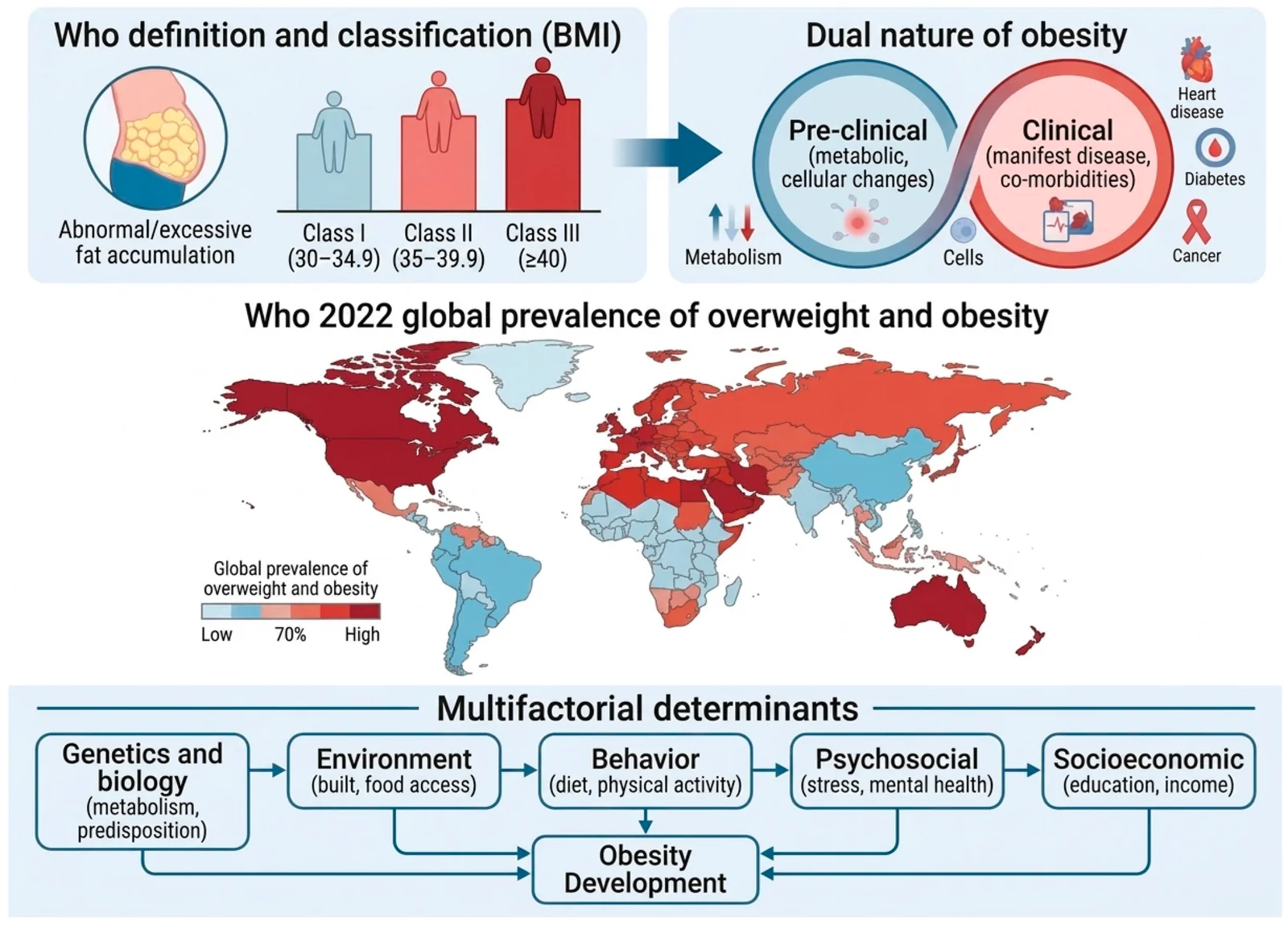
Obesity: A Global Health Challenge of the 21st Century. This infographic illustrates obesity as a multifactorial chronic disease, featuring WHO Body Mass Index (BMI) classifications (Class I: 30–34.9 kg/m^2^; Class II: 35–39.9 kg/m^2^; Class III: ≥40 kg/m^2^), the distinction between preclinical (metabolic/cellular changes) and clinical (manifest disease with comorbidities like heart disease, diabetes, cancer) stages—blue upward and red downward arrows indicate worsening metabolic responses in the transition from preclinical to clinical obesity, 2022 global prevalence maps and trends key determinants (genetics/biology, environment, behavior, psychosocial, socioeconomic). The image was generated using Inner AI version 3.0, with the Nano Banana Pro image-generation model, accessed on 5 March 2026. The final image was reviewed and edited by the authors to ensure scientific accuracy on 20 August 2026.

Global prevalence data underscore the scale of the obesity crisis. In 2022, approximately 2.5 billion adults were overweight, including 890 million with obesity. These figures represent 43% and 16% of the global adult population, respectively, reflecting a substantial increase from 1990, when about 25% of adults were overweight. Regional disparities are pronounced (Figure 1), with prevalence ranging from 31% in Southeast Asia and Africa to 67% in the Americas [5]. Trends among children and adolescents are similarly concerning. By 2024, an estimated 35 million children under five were overweight. In the 5–19 age group, over 390 million were overweight and 160 million were obese, representing a fourfold increase in obesity prevalence since 1990, from 2% to 8%. This trajectory is evident in both high-income and low- and middle-income countries, illustrating the global spread of a condition previously concentrated in affluent societies [6]. Projections from the World Obesity Atlas 2025 suggest that by 2035, over half of the global population will be overweight (BMI 25–29.9) or obese (BMI 30 or higher), with the most pronounced increases anticipated among children and adolescents [7]. For context, in 2020, approximately 764 million adults and 390 million children were living with obesity. Current trends indicate these numbers may double within the next two decades, primarily due to changes in dietary patterns, rapid urbanization, and more sedentary lifestyles [8]. In low- and middle-income countries, the health burden has resulted in a double burden of malnutrition: the simultaneous presence of undernutrition (insufficient nutrient or energy intake) and obesity (excessive body fat with adverse health effects) within the same population, household, or individual [9]. Addressing this challenge demands interventions that extend beyond traditional nutritional strategies.

The mortality burden of obesity is considerable, with approximately 4.7 million deaths annually attributed to excess body weight, primarily through cardiovascular disease, type 2 diabetes, and obesity-related cancers [10]. Epidemiological evidence demonstrates a J-shaped association between BMI and mortality risk, with all-cause mortality rising sharply above a BMI of 35 kg/m^2^ [11]. Beyond premature mortality, obesity reduces life expectancy by an estimated 8 to 10 years in severe cases [12] and significantly impairs quality of life through chronic pain, limited mobility, and increased prevalence of depression and anxiety [13].

The etiology of obesity is multifactorial, involving genetic, epigenetic, environmental, behavioral, and psychosocial determinants. Twin, family, and adoption studies indicate that 40% to 70% of obesity risk is heritable. Genes such as FTO and MC4R influence appetite regulation, energy expenditure, and fat metabolism [14]. Epigenetic modifications, including DNA methylation and histone remodeling, both shaped by diet, gut microbiota, and early developmental factors, also contribute to individual susceptibility [15]. Ecological determinants are equally influential. Rapid urbanization, widespread availability of ultra-processed foods, and increasingly sedentary lifestyles characterize the obesogenic environment. This concept encompasses the social, cultural, and economic factors that promote excessive caloric intake and reduced physical activity [16]. Behavioral and psychosocial contributors further exacerbate obesity risk. Insufficient sleep, chronic stress, and psychiatric disorders such as depression and anxiety are independently linked to weight gain, partly through dysregulation of the hypothalamic–pituitary–adrenal axis, elevated cortisol levels, and increased visceral fat accumulation [8,17,18]. Recently, the gut microbiota has emerged as an independent metabolic determinant. Alterations in the Firmicutes-to-Bacteroidetes ratio, representing the balance between two predominant bacterial phyla, have been associated with increased energy extraction from dietary substrates and the chronic low-grade inflammation characteristic of obesity [2,19,20].

Obesity is increasingly recognized as a systemic metabolic disorder in which chronic oxidative stress contributes to adipose tissue dysfunction, insulin resistance, mitochondrial impairment, and persistent low-grade inflammation. Consequently, antioxidant-based interventions have emerged as promising complementary therapeutic strategies. Although several reviews have explored the role of oxidative stress in obesity, these studies have primarily focused on the pathophysiological mechanisms of disease development and the roles of endogenous and exogenous antioxidant systems [21,22,23,24,25]. In contrast, a comprehensive synthesis specifically addressing antioxidant-based therapeutic strategies across broader obesity populations remains lacking. Therefore, the present review aims to critically summarize the current evidence on antioxidant therapies for obesity, discussing their mechanisms of action, preclinical and clinical efficacy, translational challenges, limitations, and future therapeutic perspectives. By shifting the focus from disease pathophysiology to therapeutic intervention, this review complements the existing literature and highlights current opportunities and challenges for the clinical application of antioxidant-based therapies.

## 2. Effects of Obesity on Reactive Oxygen Species (ROS) Production and Adipose Tissue Inflammation

### 2.1. Adipose Tissue Expansion and Inflammatory Remodeling

Adipose tissue expansion beyond its physiological storage capacity is accompanied by profound structural and functional remodeling [26]. Excessive lipid accumulation promotes adipocyte hypertrophy and, when the storage capacity of adipocytes is exceeded, adipocyte stress and cell death [27]. These processes increase the release of free fatty acids and danger-associated molecular signals into the local environment, promoting immune cell recruitment infiltration [28]. In parallel, adipokine secretion becomes dysregulated, with increased production of pro-inflammatory mediators and reduced anti-inflammatory signaling, contributing to the establishment of chronic low-grade inflammation [29]. Importantly, visceral adipose tissue appears to be particularly susceptible to oxidative and inflammatory remodeling, with greater accumulation of oxidized lipids and proteins compared with subcutaneous adipose tissue [30].

Inflammatory remodeling of adipose tissue is closely interconnected with oxidative stress [31]. Excess nutrient availability increases metabolic flux through mitochondrial oxidative pathways and promotes the generation of reactive oxygen species (ROS) [32]. In turn, ROS can activate redox-sensitive inflammatory pathways, including nuclear factor kappa B (NF-κB), thereby increasing the production of cytokines such as tumor necrosis factor-alpha (TNF-α) [24]. TNF-α and other inflammatory mediators further impair insulin signaling and contribute to the maintenance of metabolic dysfunction [33]. Thus, oxidative stress and inflammation should not be considered independent processes in obesity but rather interconnected components of a self-reinforcing metabolic network.

Lipid peroxidation provides an additional molecular link between adipose tissue oxidative stress and inflammation [30]. Peroxidation of polyunsaturated fatty acids generates reactive lipid aldehydes, particularly 4-hydroxynonenal (4-HNE) and malondialdehyde (MDA). These electrophilic products can modify proteins through carbonylation of lysine, histidine, and cysteine residues, potentially disrupting insulin signaling, lipid metabolism, and mitochondrial respiration [34]. Consequently, lipid-derived oxidative products may amplify both cellular dysfunction and inflammatory signaling within expanding adipose tissue [35,36,37].

### 2.2. Mitochondrial Dysfunction and ROS Generation

Mitochondria represent a major intracellular source of ROS and are particularly vulnerable to nutrient excess [38]. During obesity, increased availability of fatty acids and other energy substrates imposes a high metabolic demand on mitochondria, potentially exceeding their oxidative capacity and compromising mitochondrial homeostasis [39]. Mitochondrial alterations accompanied by increased ROS accumulation have been documented in skeletal muscle and brain tissues of high-fat diet (HFD)-fed mice, as well as in the brain and plasma of diet-induced obese rats [33,35,40,41,42,43,44,45].

The mitochondrial electron transport chain (ETC), particularly complexes I and III, constitutes an important source of superoxide generation. Under conditions of altered substrate availability or impaired electron transfer, electron leakage from the ETC can increase superoxide production [38]. In addition, pyruvate dehydrogenase and the xanthine dehydrogenase/oxidoreductase system can contribute to hydrogen peroxide (H_2_O_2_) generation during conditions of increased metabolic and lipogenic activity [46]. Endoplasmic reticulum oxidoreductin 1 (ERO1) represents another source of H_2_O_2_, generating this ROS during protein folding and secretion and thereby contributing to the oxidative burden associated with metabolic stress [47].

The magnitude of mitochondrial ROS production is highly dependent on the metabolic substrate and respiratory state [48]. Under basal NADH-linked respiration through complex I, only a small fraction of consumed oxygen is converted into superoxide. However, succinate-driven respiration through complex II can promote reverse electron transport toward complex I, markedly increasing mitochondrial ROS production [49]. Consistent with this mechanism, maximal mitochondrial H_2_O_2_ emission increases rapidly following exposure to an HFD in rats, demonstrating that dietary nutrient excess can rapidly alter mitochondrial redox homeostasis [50].

Mitochondrial quality control is also impaired during obesity [51]. Reduced mitochondrial biogenesis and alterations in mitochondrial dynamics have been observed in adipose tissue and other metabolically active organs [39]. In particular, downregulation of mitofusin 2, an important regulator of mitochondrial fusion, has been associated with increased H_2_O_2_ production and impaired metabolic function [33]. These alterations may establish a positive feedback loop in which mitochondrial dysfunction increases ROS generation, while excessive ROS further damages mitochondrial proteins, lipids, and respiratory function.

### 2.3. NADPH Oxidases and Other Sources of Oxidative Stress

Although mitochondria are an important source of ROS, obesity-associated oxidative stress cannot be attributed exclusively to mitochondrial dysfunction. NADPH oxidases (NOX enzymes) constitute another major source of ROS in adipose tissue and other metabolic organs [52]. Increased NOX activity has been associated with obesity, elevated circulating fatty acids, altered adipokine secretion, and adipose tissue inflammation [36,53,54,55,56].

Early studies demonstrated that adipose tissue can represent an important source of circulating ROS and that NADPH oxidase activation is associated with increased fatty acid concentrations and adipocytokine dysregulation. Notably, these alterations were observed in obese individuals without diabetes, suggesting that adiposity itself can contribute to oxidative stress independently of hyperglycemia [54].

Among the NOX isoforms, NOX4 appears to have a particularly complex role in adipose tissue redox regulation. NOX4 is expressed in several intracellular compartments, including the endoplasmic reticulum, nucleus, and mitochondria of adipocytes, and can contribute to H_2_O_2_ production under conditions of elevated glucose and saturated fatty acids [55]. However, its role is not exclusively detrimental. Adipocyte-specific NOX4 deficiency was shown to attenuate adipose tissue inflammation and delay the development of insulin resistance in mice fed a high-fat, high-sucrose diet, whereas global NOX4 knockout was associated with accelerated HFD-induced insulin resistance [53]. These apparently divergent findings highlight the importance of cellular context, tissue specificity, and the distinction between physiological and pathological ROS signaling [57].

NOX2 represents another relevant source of ROS [58]. Although NOX2-derived ROS may contribute to metabolic impairment in obesity and type 2 diabetes, ROS generated by this enzyme can also participate in physiological adaptations to exercise [59]. Therefore, the contribution of NADPH oxidases to obesity should be interpreted in a context-dependent manner rather than assuming that increased ROS production is invariably pathological [60].

### 2.4. Impaired Antioxidant Defenses and Redox Imbalance

The increase in ROS production associated with obesity is accompanied by alterations in endogenous antioxidant defenses [61,62]. During adipose tissue expansion, the activities of several antioxidant enzymes, including superoxide dismutase (SOD), catalase (CAT), glutathione peroxidase (GPx), glutathione S-transferase A4, peroxiredoxin 3, and GPx4, have been reported to decrease, whereas NADPH oxidase activity increases [22]. The resulting imbalance between ROS generation and antioxidant capacity favors sustained oxidative stress [25,54,63,64].

The reduction in antioxidant defenses may further increase susceptibility to oxidative damage in metabolically active tissues [21]. Excess ROS can promote lipid and protein oxidation, impair mitochondrial respiration, and interfere with insulin signaling. In obesity, these effects are particularly relevant in adipose tissue, skeletal muscle, and liver, where oxidative stress interacts with excess free fatty acids, inflammation, and impaired mitochondrial biogenesis to promote systemic metabolic dysfunction [65].

Importantly, experimental evidence suggests that the relationship between antioxidant capacity and metabolic health is not necessarily linear. For example, adipocyte-specific antioxidant enzyme overexpression does not invariably protect against obesity-associated metabolic dysfunction [66]. In high-fat diet (HFD)-fed mice, overexpression of catalase and SOD1 promoted adipose tissue expansion while reducing ectopic lipid deposition, whereas glutathione depletion impaired adipose expansion and aggravated ectopic lipid accumulation and insulin resistance [67,68,69,70]. These findings suggest that ROS levels must be considered within a physiological range and that excessive suppression of redox signaling may be metabolically detrimental.

### 2.5. Physiological Versus Pathological ROS Signaling in Obesity

An important consideration in understanding obesity-associated oxidative stress is that ROS are not exclusively harmful molecules [60]. At physiological concentrations, ROS—particularly H_2_O_2_—function as signaling mediators involved in cellular adaptation, adipogenesis, insulin signaling, and mitochondrial metabolism [71]. Therefore, the therapeutic objective should not necessarily be the complete elimination of ROS but rather the restoration of redox homeostasis and prevention of sustained oxidative damage.

A physiological increase in intracellular H_2_O_2_ appears to be required for adipocyte differentiation, whereas experimental attenuation of ROS with agents such as N-acetylcysteine (NAC), cerium oxide nanoparticles, or luteolin can inhibit adipogenesis or lipid accumulation, at least partly by interfering with adipogenic and lipogenic programs [72,73,74]. Similarly, mitochondrial ROS generated in response to adrenergic stimulation or genetic manipulation can contribute to thermogenic respiration and activation of thermogenesis-related genes [75,76,77]. These observations demonstrate that ROS may have beneficial or adaptive functions depending on their concentration, cellular localization, and duration of exposure.

The biological effects of individual ROS-generating systems further support this concept. Although NOX4 can contribute to oxidative stress under conditions of metabolic overload, global NOX4 deletion has paradoxically been associated with accelerated insulin resistance in some experimental settings [53,55,57]. Likewise, MnSOD overexpression in HFD-fed mice promoted obesity and insulin resistance, whereas MnSOD deletion triggered compensatory increases in mitochondrial biogenesis, oxygen consumption, and insulin sensitivity [40,78,79]. Such findings challenge the concept that increasing antioxidant capacity or indiscriminately suppressing ROS will invariably improve metabolic outcomes.

Taken together, these observations indicate that obesity-associated oxidative stress should be understood as a disruption of redox homeostasis rather than simply an excess of ROS. The transition from physiological redox signaling to pathological oxidative stress appears to depend on ROS concentration, duration of exposure, subcellular localization, and the balance between ROS production and antioxidant defenses. The precise threshold at which adaptive redox signaling becomes metabolically detrimental remains incompletely defined and warrants further investigation. This distinction is particularly relevant when considering antioxidant-based interventions, as therapeutic strategies that indiscriminately eliminate ROS may interfere with physiological signaling pathways and potentially produce unintended metabolic consequences.

**Figure 2:**
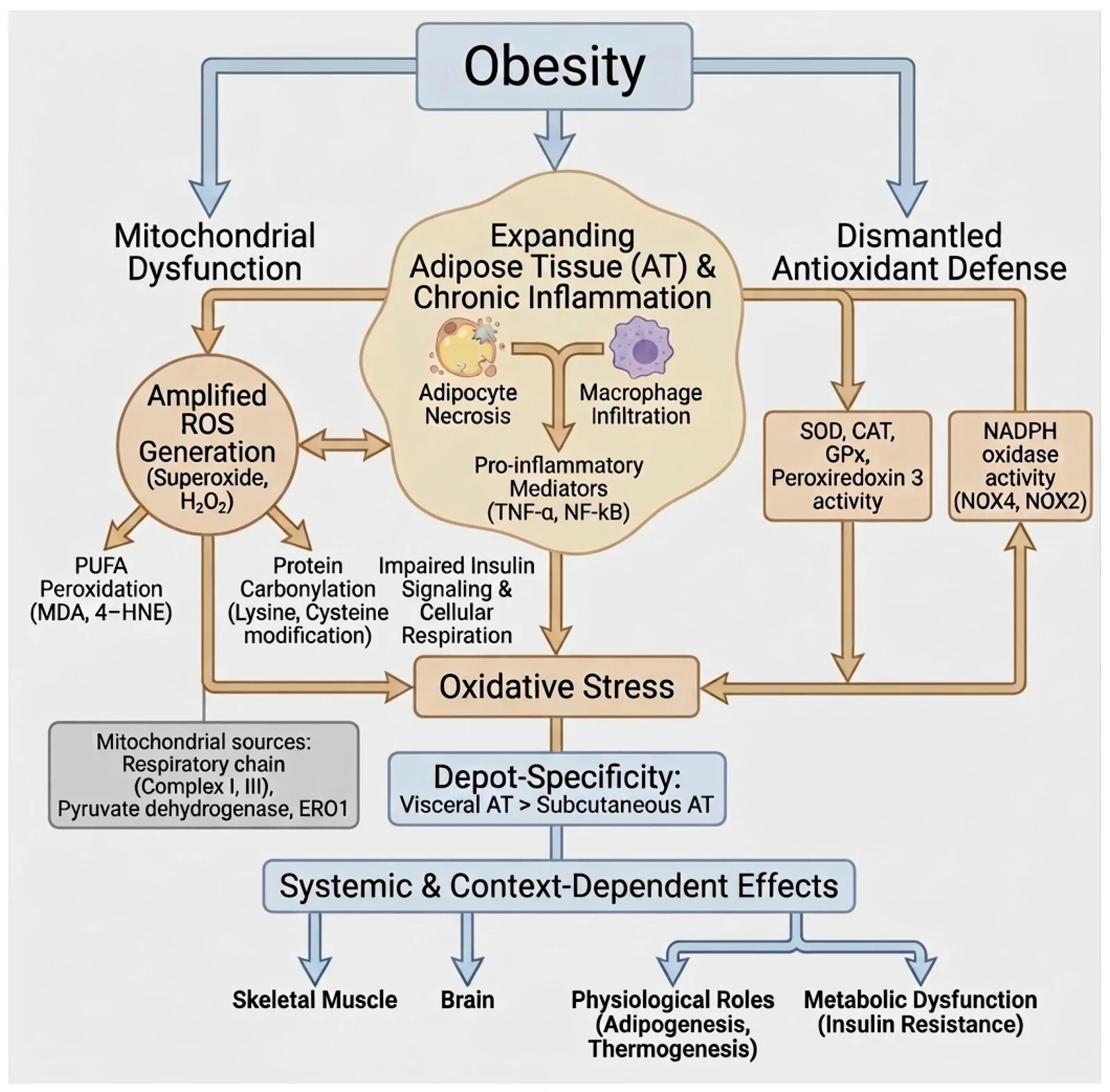
Oxidative stress and mitochondrial dysfunction in obesity-associated adipose tissue expansion. Schematic overview of obesity-driven mechanisms leading to amplified ROS generation (superoxide, H_2_O_2_ from complexes I/III, pyruvate dehydrogenase, endoplasmic reticulum oxidoreductin 1—ERO1), polyunsaturated fatty acid (PUFA) peroxidation (MDA, 4-HNE), protein carbonylation (lysine/cysteine residues), and dismantled antioxidant defenses (↓ SOD, CAT, GPx, peroxiredoxin 3; ↑ NOX2/NOX4 activity). Depot-specific effects favor visceral > subcutaneous AT, with reciprocal ROS-inflammation loops via adipocyte necrosis, macrophage infiltration, TNF-α, and NF-κB activation, perpetuating insulin resistance and metabolic dysfunction across tissues. The image was generated using Inner AI version 3.0, with the Nano Banana Pro image-generation model, accessed on 5 March 2026. The final image was reviewed and edited by the authors to ensure scientific accuracy on 20 August 2026.

Those findings are summarized in Figure 2 and show that ROS production and clearance in adipose tissue are highly context-dependent. The threshold at which redox signaling shifts from physiologically useful to metabolically damaging is not completely defined and warrants dedicated investigation.

## 3. Preclinical Approach

### 3.1. Vitamins

#### 3.1.1. Vitamin A

Vitamin A and its metabolites have shown consistent antioxidant and anti-obesity activity across multiple in vivo models. Maternal supplementation with beta-carotene during late gestation and lactation reduced offspring susceptibility to diet-induced obesity in adulthood, attenuating fat depot mass, adipocyte hypertrophy, and hepatic steatosis in mice [80]. In HFD-fed or genetically obese mice (ob/ob and db/db), retinol levels fall markedly in liver, lung, pancreas, and kidney despite adequate circulating vitamin A, a decline accompanied by reduced retinoic acid receptor expression (RARalpha, RARbeta2, RARgamma) and downregulation of retinol-binding proteins. Weight loss largely reverses these deficits [81]. Administration of all-trans retinoic acid (RA) to obese mice induces weight loss, improves insulin sensitivity, and upregulates genes involved in fatty acid oxidation and lipolysis through PPARbeta/delta and RAR signaling [82].

An in vitro study has helped to clarify the molecular basis for these observations. All-trans RA represses 3T3-L1 preadipocyte differentiation by downregulating PPARγ and fatty acid-binding proteins, while concurrently enhancing the expression of oxidative metabolism and mitochondrial biogenesis genes [83]. In adipocyte development and browning models, retinoid signaling can promote the acquisition of thermogenic features and increase UCP1 expression. These effects have been linked to RAR-dependent transcriptional mechanisms, whereas the specific contributions of progenitor-cell commitment, PRDM16 activation, p38MAPK signaling, and adipose vascularization remain model- and context-dependent [76]. These data suggest that vitamin A metabolites do more than scavenge ROS; they actively remodel redox-sensitive gene networks and influence adipose tissue plasticity.

Several caveats should be considered. The metabolic effects of vitamin A and retinoids depend on dose, duration, formulation, baseline vitamin A status, and experimental model; accordingly, effects on adiposity and lipid metabolism may vary [76,84]. Although lower circulating carotenoid concentrations and altered retinol-related biomarkers are associated with obesity in humans, causality remains uncertain [85,86]. Further studies should optimize carotenoid delivery systems, including encapsulated formulations, assess long-term safety, and clarify whether their metabolic effects arise from direct ROS scavenging or indirect modulation of adipogenesis, inflammation, and energy expenditure [76,87].

#### 3.1.2. Vitamin B

A micronutrient formulation combining folate, vitamins B6 and B12, choline, betaine, and zinc prevented HFD-induced adiposity and improved glucose homeostasis in male rats, alongside a marked increase in hepatic glutathione content and reduced hepatic triglyceride accumulation. The proposed mechanism involves redirecting one-carbon intermediates toward the transsulfuration pathway, with vitamin B6 as a cofactor, thereby enhancing glutathione synthesis and reinforcing redox buffering. This was associated with altered expression of lipid-metabolism genes, including increased CPT1A expression consistent with enhanced fatty acid beta-oxidation, and supports a model in which combined methyl-donor and antioxidant micronutrients attenuate HFD-driven oxidative stress and insulin resistance through glutathione-dependent suppression of redox-sensitive metabolic dysfunction [63].

Pyridoxine supplementation attenuated renal injury in a long-term HFD rat model by restoring antioxidant enzyme activity, reducing lipid peroxidation, and downregulating AGE-RAGE signaling, with downstream improvements in insulin signaling and reduced apoptosis. The study documented activation of the Nrf2/HO-1 axis and suppression of NOX4/AGE-RAGE/PKC alpha-driven ROS generation, alongside partial recovery of PI3K/Akt and PKC zeta components of insulin signaling. These effects correlated with decreased renal TNF-alpha and iNOS levels and lower tubular injury markers (NGAL), suggesting that pyridoxine exerts renal protection in obesity primarily via antiglycative and Nrf2-mediated mechanisms that intersect inflammatory and insulin signaling pathways [88].

In a sucrose-induced metabolic syndrome rat model, pyridoxal phosphate (PLP) administration produced anti-obesity and hepatorenal protective effects, mediated by increased total glutathione, upregulated glyoxalase-1 activity, and suppressed hepatic NF-kB expression. Functionally, PLP reduced hepatic lipid accumulation, normalized transaminase elevations, and lowered systemic inflammatory indices. These data support the concept that vitamin B6 derivatives act through glutathione- and glyoxalase-dependent redox axes to blunt glycation- and inflammation-driven pathology in metabolic disease [89]. Across these three models, glutathione acts as the central axis through which vitamin B-based strategies exert metabolic protection. By channeling one-carbon metabolism into the transsulfuration pathway, enhancing GSH biosynthesis, and activating Nrf2-dependent responses, these interventions restore redox balance, suppress lipid peroxidation and AGE-RAGE-driven carbonyl stress, and dampen downstream inflammatory signaling. These effects consistently translate into improved insulin sensitivity, better lipid handling, and preserved tissue stability.

#### 3.1.3. Vitamin C

In an HFD mouse model of metabolic-associated fatty liver disease (MAFLD), prophylactic vitamin C (30 mg/kg), administered alone or combined with vitamin D3, markedly attenuated obesity, dyslipidemia, insulin resistance, hepatic steatosis, and inflammation. Using 16S rDNA sequencing, non-targeted metabolomics, and immunohistochemistry, Chen et al. showed that vitamin C reshaped gut microbiota composition, improved barrier integrity (ZO-1, occludin), reduced oxidative stress and endotoxemia, inhibited ileal ASBT expression, upregulated hepatic CYP7A1, FXR, and BSEP, and suppressed FAS expression. The data support a model in which vitamin C exerts anti-MAFLD and anti-obesity effects by modulating the gut-liver axis, bile acid metabolism, and redox/inflammatory homeostasis [90].

A separate investigation by Lopes et al. evaluated chronic vitamin C treatment (50 mg/kg for 21 days) in male hyperadipose rats generated by neonatal monosodium glutamate exposure. These animals displayed hypertension, elevated sympathetic tone, and increased lipid peroxidation and NBT/TBARS levels in the rostral ventrolateral medulla (RVLM). Oral vitamin C normalized mean arterial pressure, improved heart rate variability (reduced LF/HF ratio), decreased plasma lipoperoxidation, and restored RVLM antioxidant capacity. Microinjection experiments revealed that vitamin C attenuated L-glutamate-induced pressor responses, implicating central antioxidant action as a mechanism for autonomic-vascular benefit [91].

These two models highlight mechanistically complementary roles for vitamin C in obesity: peripheral modulation of gut microbiota, bile acid metabolism, and hepatic lipogenesis on one hand, and central normalization of RVLM redox status and autonomic balance on the other. The convergence of lower oxidative stress markers in both models, SOD/MDA in the gut-liver axis and NBT/TBARS in the RVLM, with improvements in metabolic and cardiovascular parameters, positions vitamin C as a pleiotropic antioxidant strategy capable of addressing the combined hepatic, metabolic, and autonomic disturbances that characterize obesity.

#### 3.1.4. Vitamin E

Kato and colleagues investigated whether tocotrienols could protect against cognitive impairment in C57BL/6 mice fed a high-fat, high-sucrose diet (HFSD). Animals on the HFSD showed impaired learning in the Morris water maze, increased brain oxidative markers, including protein oxidation and 3-nitrotyrosine, and proteomic changes, including elevated ubiquitination-related proteins and reduced neuronal structural proteins. Tocotrienol supplementation preserved learning ability and reversed most proteomic alterations without markedly reducing obesity or fat mass, suggesting that tocotrienols cross the blood–brain barrier and exert neuroprotective effects by suppressing oxidative protein damage and stabilizing proteostatic pathways [92].

An in vitro study by Imessaoudene et al. examined the effect of vitamin E on adipocytes derived from obese donors. Vitamin E treatment improved lipolysis, reduced lipid peroxidation, and restored insulin sensitivity. The key pathways modulated included ROS scavenging, antioxidant enzyme activity, and lipid droplet regulation, supporting the conclusion that vitamin E reverses obesity-induced adipocyte dysfunction through direct antioxidant effects and restoration of metabolic signaling linked to insulin and lipolytic responsiveness [93].

Across these two experimental contexts vitamin E compounds appear to act not merely as direct radical scavengers but also as modulators of redox-sensitive proteostatic and metabolic pathways. Tocotrienols preserved cognitive performance and attenuated oxidative protein damage and proteomic disruption in the brains of mice exposed to diet-induced metabolic stress, whereas vitamin E improved adipocyte lipolysis, reduced lipid peroxidation, and restored insulin responsiveness in cells derived from obese donors. Together, these findings suggest that vitamin E compounds may protect against obesity-related dysfunction through tissue-specific mechanisms involving the suppression of oxidative damage, enhancement of antioxidant defenses, stabilization of proteostatic processes, and restoration of metabolic signaling.

#### 3.1.5. Vitamin D

Chronic 25-hydroxyvitamin D administration in obese, insulin-resistant rats produced metabolic and redox benefits beyond lowering fasting glucose and HOMA-IR. Circulating adiponectin rose while TNF-alpha, IL-6, and hepatic and renal accumulation of advanced glycation end-products all declined, alongside a lower oxidative stress index and improved tissue morphology in liver, kidney, heart, and vasculature. These changes are consistent with vitamin D promoting anti-inflammatory, anti-glycative, and antioxidant programming partly through restored adipokine signaling and repression of redox-sensitive transcription factors linked to fibrosis and metabolic dysfunction [94].

Conversely, inducing vitamin D deficiency alongside a high-fructose diet markedly aggravated features of metabolic syndrome. Animals developed more severe dyslipidemia, hypertension, and obesity, with increased left ventricular and aortic wall thickness, impaired endothelium-dependent vasodilation, elevated malondialdehyde (MDA) and nitric oxide, and higher cardiomyocyte injury markers (LDH, CK-MB). These findings show that vitamin D depletion amplifies oxidative and nitrosative stress, vascular inflammation, and endothelial dysfunction, effects that converge to worsen cardiovascular and metabolic phenotypes in obesity [95].

Maternal vitamin D deficiency during gestation and lactation increased adiposity, hyperlipidemia, hyperglycemia, and systemic oxidative stress in both dams and offspring. Mechanistic analysis showed downregulation of Nrf2 and its downstream target carbonyl reductase-1 (CBR1) in the placenta, liver, and pancreas of the mothers and in the liver and pancreas of the offspring. Vitamin D supplementation reactivated the Nrf2/CBR1 axis, reduced ROS, and suppressed IL-6, IL-1beta, and TNF-alpha, highlighting an intergenerational dimension of vitamin D’s role in maintaining antioxidant defenses and preventing transmission of metabolic risk [18].

In HFD-fed rats, vitamin D also protected organs beyond the classical metabolic axis. Treatment decreased MDA and nitric oxide, increased glutathione, restored bladder histopathology, and upregulated aquaporin-1 and aquaporin-3 expression, which had been suppressed by obesity. These effects indicate antioxidant and anti-inflammatory actions extending to epithelial function and fluid homeostasis [96].

Across all these experimental models, vitamin D consistently operates as a systemic modulator of redox and metabolic homeostasis in obesity. Its sufficiency supports insulin sensitivity, adiponectin signaling, and tissue function; its deficiency amplifies oxidative stress, endothelial dysfunction, and cardiovascular remodeling; and its maternal insufficiency disrupts Nrf2-dependent antioxidant defenses across successive generations. This data reframes vitamin D not as a calcium regulator with incidental metabolic effects, but as a pleiotropic micronutrient whose adequacy is indispensable for counteracting oxidative stress, inflammation, and multi-organ dysfunction in obesity.

### 3.2. Minerals

#### 3.2.1. Zinc

In rats fed a high-calorie diet, zinc aspartate administration reduced BMI and serum glucose and insulin levels, improved liver and pancreatic histopathology, and normalized oxidative stress markers. Treated obese animals showed increased catalase and SOD activities, decreased lipid peroxidation indices (conjugated dienes, TBARS, Schiff bases), and reduced protein oxidative modifications. Pancreatic islet cross-sectional area expanded toward control values, and hepatocyte morphology improved in parallel, supporting the conclusion that zinc chelation enhances redox homeostasis, reduces lipotoxicity, and helps preserve hepatic and pancreatic structure and function [97].

Zinc deficiency combined with HFD exacerbated renal injury by suppressing the Nrf2/HO-1 antioxidant pathway while concurrently activating PI3K/Akt signaling. Mice on the high-fat/low-zinc diet showed greater ROS accumulation, worse kidney function, more fibrosis, and elevated inflammation than zinc-adequate controls. In vitro, palmitate-exposed podocytes under zinc-deficient conditions showed reduced Nrf2 nuclear translocation and increased ROS levels, effects that were worsened by Nrf2 knockdown. Adequate zinc, therefore, protects renal tissue in obesity primarily by maintaining Nrf2/HO-1 activity and preventing aberrant PI3K/Akt-mediated inflammatory signaling [98].

In a combined cadmium plus HFD model, zinc supplementation mitigated cardiomyopathy by upregulating metallothionein expression, reducing oxidative damage, and improving cardiac structure and function. Lower lipid peroxidation, reduced ROS, enhanced antioxidant enzyme activities, and lower markers of cardiac injury were the consistent findings. Metallothionein, which binds heavy metals and scavenges free radicals, appears to be the central mediator of zinc’s cardioprotective effect, countering both heavy metal toxicity and obesity-associated oxidative inflammation [99].

#### 3.2.2. Selenium

Selenium modulates lipid metabolism and oxidative stress in obesity by regulating selenoprotein activity and insulin signaling. Selenite improves adipocyte differentiation, enhances insulin receptor/AKT phosphorylation, and upregulates glutathione peroxidase 3 (GPx3). However, these benefits are attenuated under obese conditions, suggesting that obesity partially impairs selenium’s capacity to restore insulin sensitivity [100]. In diet-induced hepatic injury models, selenium reduces fat accumulation, insulin resistance, and oxidative stress by activating the KEAP1/NRF2 pathway. By promoting selenoprotein synthesis, particularly SEPP1, selenium restores mitochondrial function, reduces apoptosis, and supports cellular antioxidant defenses, making it a candidate strategy against obesity-associated non-alcoholic fatty liver disease [101].

Nanoparticle-based selenium delivery has expanded its therapeutic scope. In vivo studies in high-fructose-fed mice show that selenium nanoparticles improve glucose and lipid profiles, reduce systemic inflammation (lower TNF-alpha, IL-1beta, IFN-gamma; increased IL-10), and preserve organ integrity. Complementary in silico analyses corroborate the regulation of metabolic and oxidative stress pathways, supporting the use of selenium nanoparticles as multifunctional agents against fructose-induced metabolic disturbances [102].

Additional study show that selenium enhances redox homeostasis and mitigates obesity-related organ damage by modulating mitochondrial function and antioxidant gene expression, mainly through Nrf2 activation and selenoprotein-dependent redox regulation [103]. Selenium can also improve systemic glucose tolerance and protect pancreatic beta-cell function under HFD conditions, though the magnitude of benefit depends on obesity status and selenium bioavailability [104]. Evidence confirms selenium’s dual role as an antioxidant and metabolic regulator, influencing GPx activity, Nrf2 signaling, and mitochondrial protection, while highlighting that dose optimization remains key to balance protective effects with potential toxicity [105].

### 3.3. Antioxidants

#### 3.3.1. N-Acetylcysteine (NAC)

Maternal NAC supplementation during gestation has been shown to counteract the long-term programming effects of maternal obesity on offspring liver metabolism. In male offspring of obese mothers, NAC markedly reduced oxidative stress, improved antioxidant defenses, attenuated hepatic steatosis and fibrosis markers, and normalized amino acid and lipid intermediates in metabolomic profiling. Pathway analysis indicated significant FOXO-related signaling, while suppression of the IL-6/JAK2/STAT3/FOXO6 phosphorylation cascade linked reduced inflammatory input to improved hepatic redox regulation. These data indicate that maternal NAC supplementation not only replenishes glutathione pools but also exerts long-lasting epigenetic and signaling effects that reduce the transgenerational burden of obesity-induced oxidative liver injury [62].

NAC also improves insulin sensitivity in skeletal muscle of obese animals by targeting oxidative stress-dependent inflammasome activation. In HFD models, excessive ROS promoted association of thioredoxin-interacting protein (TXNIP) with the NLRP3 inflammasome, activating inflammatory cascades and impairing insulin-stimulated glucose uptake. NAC abolished ROS-dependent TXNIP-NLRP3 proximity, reduced lipid peroxidation, and restored phosphorylation of AKT and downstream insulin signaling intermediates. Normalization of glucose uptake occurred independently of weight loss, indicating that NAC acts mainly through redox-sensitive modulation of the ROS/TXNIP/NLRP3 axis rather than through systemic metabolic changes [45].

In reproductive biology, male mice on HFD showed increased testicular oxidative stress, reduced antioxidant enzyme activity, and impaired sperm parameters. NAC supplementation reduced gonadal ROS, restored glutathione-dependent antioxidant balance, and improved sperm viability. Although these local improvements, NAC failed to prevent transmission of metabolic abnormalities, including glucose intolerance and aberrant DNA methylation at IGF2/H19 and cyp19a1 loci, to offspring. This suggests that oxidative stress is only one of multiple drivers of epigenetic reprogramming and that antioxidant therapy alone is insufficient to block intergenerational propagation of obesity-related phenotypes [106].

Neuroprotective effects of NAC have been documented through suppression of ferroptosis in the hippocampus of HFD-exposed animals. Diet-induced neuronal oxidative stress, lipid peroxidation, and labile iron accumulation were accompanied by reduced glutathione and diminished GPX4 activity, hallmarks of ferroptotic cell death. NAC replenished intracellular cysteine and glutathione pools, preserved GPX4 activity, decreased lipid ROS, and prevented synaptic loss, with animals showing better memory performance plus reduced hippocampal injury. This data link antioxidant supplementation to the mitigation of obesity-induced ferroptotic pathways in the central nervous system [107].

At the cardiovascular level, NAC mitigated adverse cardiac remodeling in rodents fed a high-saturated-fat, high-sucrose diet. Cardiac hypertrophy, diastolic dysfunction, and impaired redox homeostasis were reversed by concomitant NAC supplementation, which restored myocardial glutathione balance, reduced oxidative stress markers, and improved diastolic parameters. These effects were tissue-specific: NAC did not significantly improve diaphragm contractile function, suggesting differential sensitivity of cardiac and respiratory muscles to thiol-based antioxidant therapy. The mechanistic basis points to attenuation of oxidative stress, preservation of mitochondrial redox signaling, and normalization of hypertrophic markers [108].

#### 3.3.2. Superoxide Dismutase (SOD)

SOD status tracks closely with obesity-related oxidative injury across metabolic tissues. In human and rodent NAFLD models, hepatic mitochondrial MnSOD (SOD2) is selectively reduced, particularly in males, linking lower antioxidant capacity to steatosis severity and pointing to a sex-specific redox vulnerability [79]. Population data further suggest that total blood SOD activity reflects metabolic health status, supporting its function as a marker of obesity-related derangements in young adults [109].

In HFD-fed mice, pharmacological SOD mimetics such as MnTBAP and Tempol reduced visceral adiposity, adipocyte death, and inflammatory signaling in white adipose tissue, demonstrating that excess superoxide is causal rather than merely correlative in adipose tissue pathology [31]. Adipocytes also secrete extracellular SOD (SOD3), which rises with HFD exposure and acts locally to restrain lipid dysregulation and oxidative stress, positioning SOD3 as an adipose-derived cytoprotective mediator [110].

Targeted enhancement of mitochondrial SOD2 improves insulin action in insulin-sensitive organs. Skeletal muscle-directed SOD2 overexpression ameliorated HFD-induced insulin resistance, supporting a model in which mitochondrial superoxide burden disrupts insulin signaling reversibly through the SOD2 axis [40]. These convergent findings, namely hepatic SOD2 deficiency in NAFLD, adipose benefits of SOD mimetics and SOD3, and muscle insulin-sensitizing effects of SOD2, position SOD as a unifying redox checkpoint linking obesity to downstream metabolic dysfunction.

From a translational standpoint, exogenous SOD faces pharmacokinetic limitations, including rapid clearance and gastrointestinal degradation. The most viable approaches involve PEGylated or nano- or liposomal carriers and small-molecule SOD mimetics, which improve stability and tissue exposure [111]. In preclinical models, the mimetic tempol improved insulin sensitivity and reduced oxidative stress in obese Zucker rats [112], while MnTBAP reduced visceral adiposity and inflammatory signaling in HFD-fed mice [31]. Consistent human efficacy, however, remains to be established and will depend on optimized formulations and medication strategies [64].

#### 3.3.3. Glutathione

In a dexamethasone-driven insulin resistance model that recapitulates key features of metabolic syndrome, the SOD-glutathione axis operates as the primary brake on oxidative damage. SOD catalyzes the dismutation of superoxide to H_2_O_2_, while reduced glutathione (GSH), acting through glutathione peroxidases, reduces peroxides and maintains protein thiols in their reduced state. Dexamethasone disturbs this balance, but treatment with the nitric oxide donor molsidomine restores antioxidant capacity, raising hepatic and aortic SOD activity and GSH levels while lowering MDA. These biochemical improvements co-occur with reductions in fasting glucose, insulin, and HOMA-IR, suggesting that supporting the SOD-glutathione axis interrupts ROS-driven propagation of inflammatory cascades (including HMGB1/JAK1/STAT3 and NF-kB) and thereby improves insulin signaling and tissue morphology [113].

In diet-induced NAFLD, hepatic glutathione (GSH) availability is an important determinant of the capacity of hepatocytes to withstand oxidative injury. Evidence from studies of severe calorie restriction and inadequate protein intake suggests that insufficient protein availability may aggravate oxidative stress and inflammatory signaling, whereas nutritionally adequate weight-loss interventions mitigate these risks. This relationship is biologically plausible because cysteine availability can limit GSH synthesis and influence cellular redox balance, including the GSH/GSSG ratio. Accordingly, nutritional strategies for NAFLD should aim to preserve adequate protein and sulfur-amino-acid availability while reducing energy intake, rather than emphasizing caloric restriction alone [114].

In obese muscle, sustained ROS overproduction depletes the GSH pool (decreased GSH, increased GSSG), driving lipid peroxidation and contractile dysfunction. Both exercise and diazoxide, a KATP channel opener, attenuated oxidative stress and restored the GSH/GSSG ratio, directly improving contractile force. Pharmacologically, three complementary strategies emerge from this model: substrate supplementation (cysteine donors to sustain GSH biosynthesis), Nrf2 activation to enhance glutathione-related enzyme expression, and channel modulators that protect GSH pools by reducing mitochondrial ROS [115].

#### 3.3.4. Coenzyme Q-10 (CoQ10)

In diet-induced obese mice, CoQ10 lowered hepatic oxidative burden and inflammatory gene expression without substantially affecting body mass, a profile consistent with primary redox modulation rather than weight loss per se [36]. In steatotic liver, CoQ10 activated AMPK and repressed lipogenesis while promoting beta-oxidation; these metabolic shifts were accompanied by reductions in lipid peroxidation and ROS signaling in both HFD mice and palmitate-challenged hepatocytes, pointing to mitochondrial redox stabilization as the upstream driver [116]. In high-fat/high-fructose insulin-resistant rats, oral CoQ10 decreased MDA, restored glutathione status, and improved PI3K-dependent insulin signaling, a classical pattern of ROS suppression leading to membrane and mitochondrial protection and, consequently, better metabolic signaling [117].

Animal and cellular studies support a mechanistic model in which coenzyme Q10 (CoQ10), an essential mitochondrial electron-transport cofactor and lipid-soluble antioxidant, attenuates oxidative stress by limiting superoxide-associated lipid peroxidation and oxidative protein damage. CoQ10 may also preserve endogenous antioxidant capacity, including glutathione-dependent defenses and antioxidant-enzyme activity, while modulating redox-sensitive metabolic and inflammatory signaling in hepatic and adipose tissues. In this framework, improvements in insulin sensitivity and hepatic steatosis may arise, at least in part, from reduced mitochondrial oxidative stress and improved redox homeostasis rather than from unrelated pharmacological actions [118].

#### 3.3.5. Alpha-Lipoic Acid (ALA)

In obese rodents, ALA activates AMPK and suppresses hepatic lipogenesis while promoting beta-oxidation, changes tightly coupled to redox normalization and demonstrating that the metabolic benefits are mechanistically downstream of oxidative stress control [119]. In HFD-fed rats, ALA reduced lipid peroxidation (MDA/LOOH) and nitrosative stress (NOX/peroxynitrite/S-nitrosothiols) while improving glutathione redox status in both visceral and subcutaneous fat, with more pronounced effects in visceral depots, linking improved adipose inflammation and apoptosis markers to restored redox buffering [120].

ALA also attenuates macrophage infiltration and TLR4/NF-κB signaling in visceral adipose tissue, linking its anti-inflammatory actions to oxidative stress containment [37]. In the vasculature, ALA prevents endothelial dysfunction and vascular oxidative stress in obese rats, consistent with systemic ROS control as a unifying mechanism [121]. Across cell and animal models of obesity, ALA’s benefits consistently map onto mitigation of oxidative and nitrosative stress via AMPK activation, glutathione support, and suppression of NOX/NF-kB axes, normalizing redox-sensitive metabolic and inflammatory pathways in liver, adipose, and vascular tissues.

### 3.4. Amino Acids

#### 3.4.1. L-Carnitine

In high-fructose-fed rats, L-carnitine (LC) reduced skeletal muscle lipid peroxidation and restored antioxidant defenses in parallel with enhanced insulin sensitivity, linking improved redox balance to protection against diet-driven metabolic dysfunction [35]. In hereditary hypertriglyceridemic rats, LC alleviated hepatic steatosis, improved mitochondrial oxidative capacity, and lowered oxidative stress, demonstrating that obesity- and diet-associated ROS generation can be attenuated through LC-mediated mitochondrial control [122]. In mice on a methionine-choline-deficient diet, a model of steatohepatitis, LC attenuated both hepatic and cardiac injury, suppressing oxidative stress and fibrosis [123]. Complementary evidence in HFD-induced NASH shows that LC increased beta-oxidation and upregulated hepatic antioxidant systems, thereby preventing progression of fatty liver disease despite continued dietary stress [124].

At the molecular level, LC exerts antioxidant action by activating the Nrf2/Keap1 pathway, including modulation of glutathione-dependent defenses [125]. In metabolic dysfunction, L-carnitine supplementation has been associated with reduced lipid peroxidation, as indicated by lower MDA levels, and enhanced antioxidant defenses, particularly SOD activity [126]. These biochemical advances are paralleled by functional improvements: reduced adipose inflammation, ameliorated insulin resistance, and improved vascular reactivity. The dual function of LC as a mitochondrial substrate shuttle and antioxidant modulator allows it to intervene at both ROS generation and antioxidant defense levels, making it a pleiotropic candidate for obesity-related complications.

#### 3.4.2. Beta-Alanine

Beta-alanine supports antioxidant protection in obesity primarily as the rate-limiting precursor of carnosine, a dipeptide that quenches reactive carbonyl and oxygen species generated under HFD stress. A direct comparison of oral beta-alanine versus carnosine in an HFD rat model showed that while both raised muscle carnosine, only carnosine dosing increased plasma carnosine, and this was the arm that attenuated early lipoxidation (reduced circulating AGE/ALE markers) and inflammatory signaling. These data point to the circulating carnosine pool, rather than muscle content alone, as the primary antioxidant effector under diet-induced metabolic stress [127].

Mechanistically, the beta-alanine-to-carnosine pathway counters obesity-related oxidative injury by trapping lipid peroxidation-derived aldehydes such as 4-HNE and limiting downstream protein carbonylation and NF-kB/MAPK activation [128]. Carnosine directly scavenges reactive species and 4-HNE adducts in metabolically relevant tissues, preserving protein function and mitochondrial integrity under glucolipotoxic conditions [129]. Boosting carnosine availability through beta-alanine supplementation lowers MDA and advanced glycation/lipoxidation end-products, and mitigates insulin-resistance-linked inflammation in liver and muscle, an effect further supported by carnosine analog studies showing improved cardiometabolic phenotypes under high-fat/high-sucrose stress [130].

#### 3.4.3. Taurine

In HFD-fed rodents, taurine treatment increases the activity and expression of SOD, GPx, and CAT, reducing ROS accumulation and MDA levels in liver and adipose tissue [131]. In mildly obese ICR mice on HFD, taurine downregulated PPARgamma, C/EBPalpha, C/EBPbeta, and aP2 expression in white adipose tissue while promoting mitochondrial biogenesis and thermogenic gene expression, including PGC-1alpha and UCP1, in brown adipose tissue and beige adipocytes, thereby increasing energy expenditure and reducing lipid-induced ROS generation [132].

Taurine also modulates inflammation in a manner closely associated with oxidative stress. In obese preclinical models, HFD induces adipose macrophage infiltration, predominantly pro-inflammatory M1 polarization, and upregulates TNF-alpha and IL-6 through NF-kB activation, which in turn amplifies ROS via NADPH oxidases and mitochondrial dysfunction. Taurine supplementation reduces macrophage infiltration, shifts polarization toward the M2 phenotype, dampens NF-kB signaling, and lowers inflammatory cytokine expression thereby interrupting the reciprocal amplification of redox imbalance and metabolic inflammation in obesity [133]. Additional molecular effects reported in experimental models include improvements in hepatic injury markers, attenuation of lipid peroxidation, enhancement of antioxidant defenses, and modulation of genes involved in lipid metabolism, including PPARα, CPT-1, and FAS. Taurine may also preserve mitochondrial structure and bioenergetic function by limiting oxidative damage and excessive ROS generation, thereby supporting metabolic homeostasis [134].

### 3.5. Hormones

#### Melatonin

In HFD-fed mice, melatonin modulates signaling pathways at the intersection of energy metabolism and redox control by reducing adipocyte hypertrophy, hepatic steatosis, inflammation, and ROS accumulation while upregulating SOD and CAT expression and reducing lipid peroxidation markers [135]. In Zucker diabetic fatty rats, melatonin administered at increasing doses enhanced brown adipose tissue (BAT) thermogenic capacity in a dose-dependent manner, restored mitochondrial dynamics (increased OPA1, decreased DRP1), elevated UCP1 levels, and reduced body weight and fat mass [75].

Melatonin additionally exerts anti-lipogenic, pro-lipolytic, and anti-inflammatory effects in white adipose tissue. Supplementation in obese mice reduced lipogenesis rates, increased lipolytic gene expression, lowered serum triglycerides and LDL, and reduced macrophage infiltration and crown-like structures in WAT, effects accompanied by decreased oxidative damage markers and increased adipocyte oxygen consumption [136]. Taken together, these findings highlight melatonin as a key metabolic regulator that combats obesity by improving mitochondrial dynamics, reducing inflammation, and mitigating oxidative stress.

## 4. Clinically Used Pharmacological Agents with Potential Indirect Effects on Oxidative Stress

### 4.1. Fenofibrate

In HFD mice, fenofibrate decreases adipocyte size, improves systemic glucose tolerance, and upregulates fatty acid oxidation programs consistent with adipose PPARalpha activation [137]. Chronic treatment also drives beigeing of subcutaneous white adipose tissue, inducing UCP1, PGC-1alpha, PRDM16, and other thermogenic genes, increasing energy expenditure and reducing lipid-driven oxidative burden [138].

Hepatic outcomes additionally demonstrate fenofibrate’s redox coupling. In HFD-induced NAFLD, fenofibrate preserves peroxisomal fitness through PPARalpha-dependent biogenesis and fatty acid oxidation, attenuating lipid accumulation and liver injury while reducing oxidative stress markers including 4-HNE, nitrotyrosine, and 8-oxo-dG. These effects are absent in Pparalpha-/- mice, confirming mechanism specificity [91]. In high-fat/high-fructose rat models, fenofibrate improves glycemic status and lipid profiles while restoring antioxidant defenses (elevated SOD activity; reduced MDA) [139].

Beyond liver and adipose tissue, fenofibrate protects against obesity-related renal injury. Delayed treatment in HFD-induced nephropathy activates AMPK and enhances autophagy, processes that support mitochondrial quality control and limit ROS-driven damage, leading to corresponding improvements in biochemical injury markers and histology [140]. Across preclinical systems, fenofibrate counters obesity-associated dysfunction by coordinating PPARalpha-mediated lipid catabolism with thermogenic remodeling and organ-protective stress responses, thereby normalizing redox status.

### 4.2. Exenatide

Exenatide is a glucagon-like peptide-1 receptor (GLP-1R) agonist whose primary pharmacological effects include enhancing glucose-dependent insulin secretion, suppressing glucagon release, delaying gastric emptying, and reducing appetite, thereby improving glycemic control and promoting weight loss. The antioxidant effects described in experimental models are considered secondary consequences of these primary metabolic actions. In HFD-induced obese mice, exenatide lowers hepatic oxidative stress while restoring mitochondrial function and insulin sensitivity [141]. In a diet-induced steatohepatitis model, exenatide remodels hepatic mitochondrial lipid metabolism, reducing lipotoxic intermediates such as diacylglycerols and ceramides and improving tricarboxylic acid cycle flux, changes consistent with decreased ROS generation and improved bioenergetics [142]. In C57BL/6 mice with diet-induced NAFLD, exenatide delays disease progression through mitophagy-linked inhibition of the NLRP3 inflammasome, thereby reducing mitochondrial ROS-driven sterile inflammation [143]. Collectively, these findings indicate that exenatide indirectly attenuates oxidative stress by improving metabolic homeostasis, reducing lipotoxicity, promoting mitochondrial quality control, and enhancing endogenous antioxidant defenses.

### 4.3. Metformin

In HFD-induced obese mice, metformin activates the AMPK/Nrf2/HO-1 axis in liver and adipose tissue, reducing superoxide and hydrogen peroxide production while upregulating SOD2, catalase, GPx, and glutathione reductase, thereby coordinating an antioxidant response that attenuates steatosis, adipose inflammation, and systemic insulin resistance [144,145,146]. In ob/ob mice, metformin restores mitochondrial membrane potential, suppresses complex I-derived superoxide, and normalizes the GSH/GSSG ratio in skeletal muscle and liver, directly linking its insulin-sensitizing effect to mitochondrial redox stabilization [147].

Mechanistically, metformin can produce a mild inhibition of mitochondrial respiratory complex I, thereby modulating electron flow and limiting excessive mitochondrial superoxide generation while generally preserving cellular energetic homeostasis at pharmacologically relevant concentrations. This mitochondrial effect may increase the AMP:ATP ratio and activate AMPK. Activated AMPK can promote Nrf2 nuclear accumulation, enhancing antioxidant gene expression, while also suppressing mTORC1 signaling. These actions may collectively reduce oxidative and anabolic stress under nutrient-excess conditions [148]. In HFD-fed rats, metformin has been shown to improve metabolic and oxidative-inflammatory abnormalities, including the modulation of AMPK- and antioxidant-related pathways. In complementary experimental models, metformin attenuates IKK/NF-κB signaling and inflammatory cytokine production through AMPK-related mechanisms, providing a plausible link between redox regulation and adipose tissue inflammation [149,150]. This merging studies above reinforces the view that metformin acts as a systemic redox modulator, with its insulin-sensitizing and organ-protective effects mechanistically downstream of ROS containment via AMPK and Nrf2 activation.

### 4.4. Orlistat

In obese rats, orlistat significantly lowered body weight, fasting glucose, serum insulin, and triglycerides, and these whole-body metabolic improvements co-occurred with reduced hepatic and adipose lipid peroxidation, lower MDA levels, and restored antioxidant enzyme activity [151]. The mechanistic interpretation is straightforward: by reducing dietary fat absorption, orlistat lowers the flux of long-chain fatty acids reaching metabolic tissues, thereby diminishing substrate-driven mitochondrial ROS generation and lipid peroxidation cascades. It should be noted that orlistat’s antioxidant effects appear largely indirect: unlike metformin, exenatide, or ALA, orlistat does not directly activate antioxidant gene networks such as Nrf2/HO-1 or modulate AMPK. Its redox benefits are primarily a consequence of reduced lipid substrate availability rather than intrinsic pharmacological targeting of ROS pathways [152]. This distinction has practical implications, since orlistat may be less effective in settings where oxidative stress is driven by sources beyond dietary fat overload, such as fructose-driven de novo lipogenesis or mitochondrial electron-transport dysfunction.

### 4.5. Sibutramine

Unlike the pharmacological agents discussed above, sibutramine should not be regarded as a conventional antioxidant therapy. Its primary anti-obesity action is mediated predominantly by inhibition of norepinephrine and serotonin reuptake by its active metabolites, thereby enhancing central satiety signaling and reducing food intake [153]. Sibutramine is not established to act primarily through direct ROS scavenging or through activation of endogenous antioxidant defenses. Accordingly, any improvement in oxidative-stress markers should be interpreted cautiously as a possible secondary consequence of reduced energy intake, weight loss, and improved metabolic homeostasis, rather than as evidence of a primary redox-modulating mechanism [154].

The available preclinical evidence further supports a cautious interpretation of its redox effects. In adult Wistar rats treated intragastrically with sibutramine for 28 consecutive days, reductions in SOD, CAT, and GPx activities were observed in salivary gland tissue, together with depletion of total glutathione and urate and increased lipid peroxidation assessed by TBARS. These changes were accompanied by increased AKT phosphorylation, interpreted as activation of redox-sensitive PI3K/AKT signaling in response to oxidative imbalance [155]. Thus, rather than demonstrating an antioxidant effect, these findings suggest that sibutramine may adversely affect redox homeostasis under certain experimental conditions.

The regulatory status of sibutramine also requires careful consideration. Sibutramine was initially approved by the U.S. Food and Drug Administration (FDA) in 1997 but was subsequently withdrawn from the United States market in December 2010 after clinical evidence indicated an increased risk of cardiovascular events, including myocardial infarction and stroke [156]. Similarly, the European Medicines Agency (EMA) concluded in 2010 that the benefits of sibutramine-containing medicines did not outweigh their cardiovascular risks and recommended suspension of their marketing authorizations throughout the European Union [157]. In Brazil, sibutramine remains authorized for the treatment of obesity under specific regulatory restrictions and controlled prescribing requirements [158]. Therefore, sibutramine should not be described as an FDA-approved anti-obesity drug or as a medication approved worldwide.

Within the context of antioxidant-based strategies, sibutramine is therefore best regarded as a clinically used anti-obesity pharmacological agent whose potential effects on oxidative stress are secondary to its primary effects on appetite, energy intake, and body weight, rather than as a conventional antioxidant therapy. Its inclusion in this review illustrates the importance of distinguishing between the primary pharmacological mechanisms of anti-obesity agents and secondary changes in redox homeostasis that may accompany metabolic improvement or drug-induced metabolic stress.

## 5. Emergent Therapies

### Salicylic-Acid Nitroalkene (SANA)

The salicylic acid nitroalkene derivative SANA has emerged from recent preclinical study as a structurally novel small molecule with anti-obesity effects mechanistically rooted in mitochondrial and redox biology. In HFD-fed mouse models, SANA administration reduced body weight gain, improved glucose tolerance, lowered hepatic lipid accumulation, and increased whole-body energy expenditure. Notably, these effects were maintained even under thermoneutral conditions, which effectively eliminates adrenergic-driven thermogenesis as an explanatory variable [77].

What distinguishes SANA from classical thermogenic compounds is that its metabolic effects occur independently of UCP1. This points toward alternative mitochondrial energy-dissipating pathways, most plausibly creatine-dependent substrate cycling, in which the phosphocreatine-creatine shuttle operates as an energy-wasting futile cycle that increases mitochondrial ATP turnover and respiratory chain activity without net muscle contraction. Amplifying oxidative phosphorylation flux through this mechanism reshapes intracellular redox dynamics: it increases the rate of mitochondrial electron transport and, consequently, the probability of incomplete reduction events at complexes I and III, generating superoxide as a physiological byproduct [159]. How cells manage this redox consequence, whether through adaptive upregulation of SOD2, peroxiredoxins, or Nrf2-dependent gene programs, has not yet been systematically addressed in the SANA literature and represents a critical mechanistic gap for future studies. Disentangling these contributions will be essential before SANA’s therapeutic profile can be meaningfully compared with that of established antioxidant-active anti-obesity agents.

All these preclinical approaches are summarized in Table 1, which highlights the antioxidant and redox-modulating interventions investigated in each experimental model of obesity, the redox mechanism proposed and the primary outcomes. 

## 6. Conclusions

**Figure 3:**
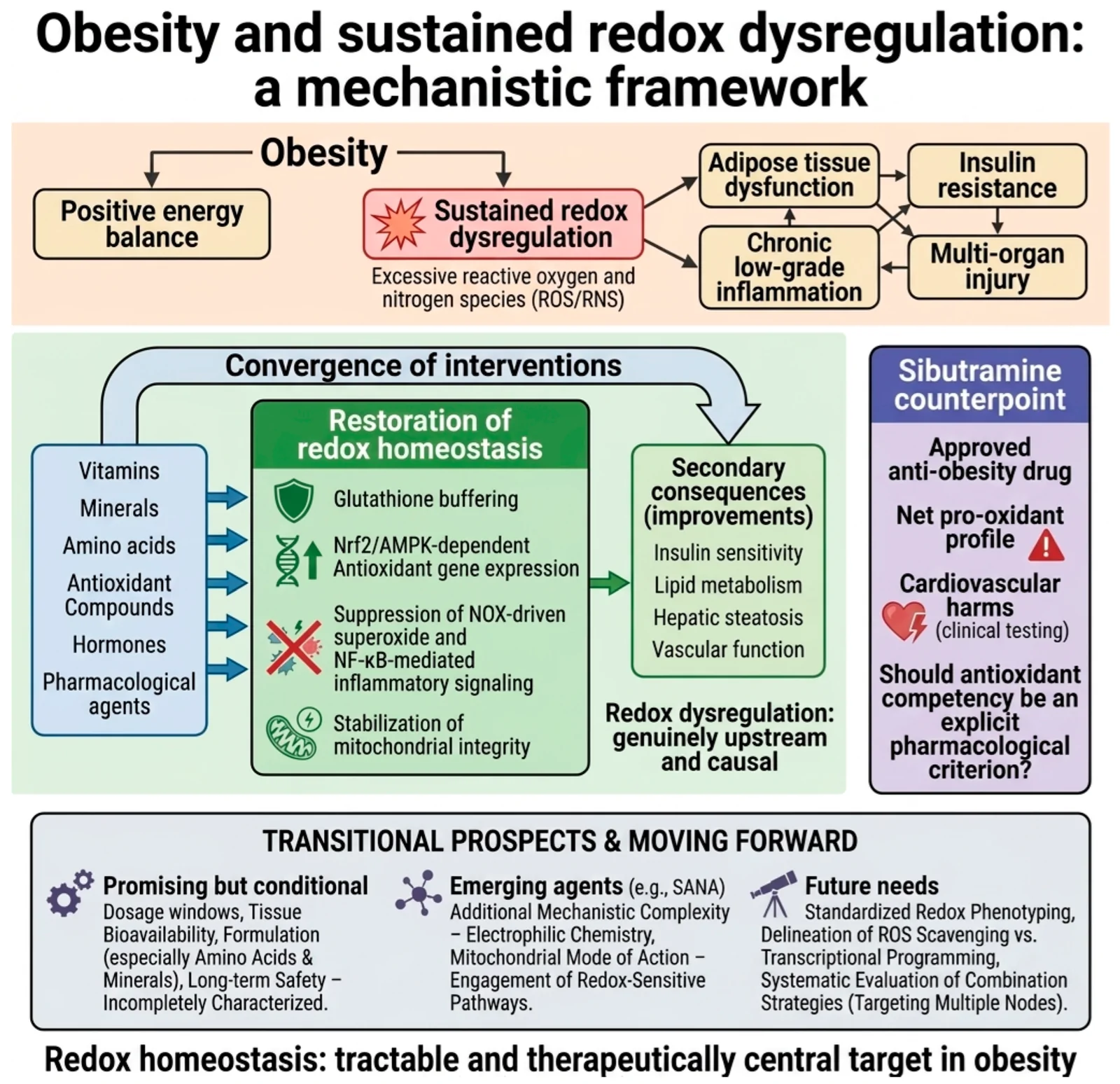
Therapeutic Convergence on Redox Homeostasis in Obesity. Orange box Schematic showing obesity-driven redox dysregulation (ROS/RNS excess) causing adipose dysfunction, insulin resistance, inflammation, and multi-organ injury. Thin black arrows indicate the cause-and-effect relationships among the factors associated with obesity. Green box: Several mechanistically distinct interventions (vitamins, minerals, antioxidants, hormones, drugs) converging via Nrf2/AMPK, glutathione, NOX/NF-κB suppression, and mitochondrial stabilization to restore redox balance and yield secondary metabolic improvements. Purple box: Sibutramine counterpoint underscores need for antioxidant competency in therapies. Grey box: A prospective frame about the management of redox status in obesity. The image was generated using Inner AI version 3.0, with the Nano Banana Pro image-generation model, accessed on 5 March 2026. The final image was reviewed and edited by the authors to ensure scientific accuracy on 20 August 2026.

Preclinical models consistently demonstrate that obesity is characterized by sustained redox dysregulation, in which excessive reactive oxygen and nitrogen species contribute to adipose tissue dysfunction, insulin resistance, chronic inflammation, and multi-organ injury. Mechanistically distinct interventions such as vitamins, minerals, amino acids, antioxidant compounds, hormones, and pharmacological agents, converge on restoring redox homeostasis. These interventions act through glutathione buffering, Nrf2/AMPK-dependent antioxidant gene expression, suppression of NOX-driven superoxide and NF-kB-mediated inflammatory signaling, and stabilization of mitochondrial integrity. Improvements in insulin sensitivity, lipid metabolism, hepatic steatosis, and vascular function observed with these interventions are largely secondary to the containment of ROS and restoration of redox balance. Thus, redox dysregulation occupies a primary, causal role in obesity-related pathophysiology. Figure 3 summarizes main findings raised in this review.

The case of sibutramine, an anti-obesity drug with a pro-oxidant profile and associated cardiovascular risks, underscores the need to consider antioxidant competency as a criterion in evaluating metabolic therapies. Current preclinical paradigms often overlook this aspect. While translational prospects are promising, they remain conditional on further research into dosage, tissue bioavailability, formulation strategies, and long-term safety, particularly for compounds with narrow therapeutic ranges. Emerging agents such as SANA introduce mechanistic complexity, as their electrophilic chemistry and mitochondrial actions may engage redox-sensitive pathways that are not fully assessed by standard antioxidant assays.

Future research should prioritize standardized redox phenotyping, clearer mechanistic distinctions between direct ROS scavenging and transcriptional antioxidant programming, and systematic evaluation of combination strategies targeting multiple nodes of the redox-metabolic network. The available preclinical evidence positions redox homeostasis as a central and actionable therapeutic target in obesity, providing a foundation for the development of more refined clinical interventions.

## 7. Scope, Search Strategy, and Limitations

This narrative review aimed to identify and synthesize preclinical evidence on the relationship between oxidative stress, redox signaling, and obesity, with particular emphasis on antioxidant and redox-modulating strategies investigated in experimental models of obesity and obesity-related metabolic dysfunction. A structured search of PubMed/MEDLINE and Scopus was conducted using combinations of terms related to obesity, oxidative stress, reactive oxygen species, redox signaling, antioxidant defenses, oxidative damage, mitochondrial dysfunction, adipose tissue inflammation, and the specific pharmacological interventions discussed in the review. Boolean operators were used to combine the search terms, and additional relevant studies were identified by screening the reference lists of key publications. The search primarily focused on studies published during the preceding five years; however, earlier studies were retained when they were necessary to establish fundamental mechanisms, provide historical context, or support well-characterized biological pathways.

Studies were considered eligible when they investigated oxidative stress, redox signaling, antioxidant defenses, mitochondrial dysfunction, or related molecular mechanisms in the context of obesity or obesity-associated metabolic disorders. Both in vitro and in vivo preclinical studies were included, particularly those evaluating antioxidant or redox-modulating interventions. Human clinical studies were excluded because the review was specifically designed to examine preclinical evidence and the molecular mechanisms underlying these strategies. Studies were also excluded when they did not directly address obesity or obesity-related metabolic dysfunction, failed to investigate oxidative or redox mechanisms relevant to the scope of the review, or lacked sufficient experimental or mechanistic information. Reviews and meta-analyses were used mainly to identify relevant primary studies and provide broader context, whereas mechanistic interpretations were based preferentially on original experimental research.

Several limitations should be considered when interpreting the evidence summarized here. Because this manuscript is a narrative rather than a systematic review, it was not registered in PROSPERO, no formal PRISMA protocol was followed, and no quantitative meta-analysis or formal risk-of-bias assessment was performed. Accordingly, the conclusions should be understood as a critical synthesis of the available preclinical literature rather than as definitive evidence of causality. The evidence base is also characterized by substantial heterogeneity in experimental models, including diet-induced, genetic, and chemically induced obesity, which reproduce different aspects of the pathophysiology of human obesity. In addition, many studies were conducted exclusively in male animals or did not clearly report the sex of the experimental subjects, limiting the assessment of sex-specific differences in redox regulation and metabolic responses. Differences in dietary composition, treatment dose, treatment duration, sample size, experimental design, and the limited use of pair-feeding protocols may further contribute to variability in the reported outcomes.

The interpretation of antioxidant interventions is complicated by their frequently pleiotropic effects. Improvements in oxidative stress markers may reflect primary pharmacological actions, but they may also occur secondarily to reduced adiposity, improved glycemic control, decreased lipotoxicity, or broader improvements in metabolic homeostasis. Changes in the gut microbiota, intestinal barrier function, and short-chain fatty acid production may also contribute to the effects attributed to antioxidant therapies. Furthermore, several compounds may act predominantly by inducing adaptive endogenous defense mechanisms, particularly through Nrf2-dependent pathways, rather than by directly scavenging reactive oxygen species. This distinction is important because physiological ROS participate in essential processes, including cellular signaling, adipogenesis, and insulin action. Therefore, therapeutic efficacy should be considered in terms of restoring redox homeostasis rather than indiscriminately suppressing ROS production. Future studies should use more standardized experimental protocols, include both sexes, apply rigorous methodological quality assessments, incorporate appropriate controls for energy intake and body-weight changes, and provide mechanistic and clinical validation to clarify the therapeutic potential of redox-modulating strategies for obesity and its metabolic complications.

## Figures and Tables

**Table 1:** Antioxidant and redox-modulating strategies investigated in preclinical models of obesity: mechanisms of action and main reported effects.

Antioxidant/ Strategy	Main Experimental Evidence/Model	Main Molecular Targets/ Pathways	Proposed Antioxidant/ Redox Mechanism	Main Reported Effects	Refs.
Vitamin A/β-carotene	HFD-fed and genetically obese mice (ob/ob, db/db); maternal supplementation	RARα, RARβ2, RARγ; PPARβ/δ; PRDM16; p38 MAPK	Modulation of redox-sensitive gene networks; promotion of oxidative metabolism and mitochondrial biogenesis; indirect regulation of adipogenesis and energy expenditure	↓ adipose mass; ↓ adipocyte hypertrophy; ↓ hepatic steatosis; ↑ insulin sensitivity; ↑ fatty acid oxidation; ↑ thermogenesis	[80,81,83]
Vitamin B complex (folate, B6, B12, choline, betaine, zinc)	HFD-fed male rats	Transsulfuration pathway; glutathione synthesis; CPT1A	Increased GSH biosynthesis and redox buffering through one-carbon metabolism	↓ adiposity; improved glucose homeostasis; ↑ hepatic GSH; ↓ hepatic triglycerides; ↑ fatty acid β-oxidation	[63,88,89]
Vitamin B6/Pyridoxine	Long-term HFD rats; sucrose-induced metabolic syndrome rats	Nrf2/HO-1; NOX4; AGE-RAGE; PKC; PI3K/Akt	Activation of endogenous antioxidant defenses; suppression of NOX4-driven ROS; reduction in glycation/carbonyl stress	↓ lipid peroxidation; ↓ renal/hepatic injury; ↓ inflammation; improved insulin signaling; ↓ hepatic lipid accumulation	[88,89]
Vitamin C	HFD mouse model of MAFLD; neonatal MSG-induced hyperadipose rats	Gut-liver axis; ZO-1/occludin; ASBT; FXR; CYP7A1; BSEP; FAS; central RVLM redox pathways	ROS reduction; improvement of intestinal barrier; modulation of microbiota and bile acid metabolism; restoration of central antioxidant capacity	↓ obesity; ↓ dyslipidemia; ↓ insulin resistance; ↓ hepatic steatosis/inflammation; ↓ lipid peroxidation; improved autonomic/cardiovascular parameters	[90,160]
Vitamin E/Tocotrienols	HFSD-fed C57BL/6 mice; obesity models with α-tocopherol/apelin	ROS scavenging; antioxidant enzymes; NF-κB; mitochondrial biogenesis; lipid-droplet regulation	Direct ROS scavenging and enhancement of endogenous antioxidant defenses; reduction in lipid peroxidation	Neuroprotection; ↓ oxidative protein damage; improved adipocyte lipolysis and insulin sensitivity; improved cardiac and mitochondrial function	[36,92]
Vitamin D/25-hydroxyvitamin D	Obese insulin-resistant rats; HFD/high-fructose models; maternal deficiency models; HFD rats	Nrf2/CBR1; adiponectin; inflammatory pathways	Antioxidant, anti-inflammatory and anti-glycative signaling; restoration of Nrf2-dependent defenses	↓ oxidative stress; ↓ inflammatory cytokines; ↑ adiponectin; improved insulin sensitivity; ↓ cardiovascular and multi-organ injury	[18,90,94,95,96]
Zinc	High-calorie diet rats; HFD/low-zinc mice; cadmium + HFD models	Nrf2/HO-1; PI3K/Akt; metallothionein	Maintenance of endogenous antioxidant defenses; metallothionein-mediated ROS protection; reduction in lipid peroxidation	↓ BMI and glucose; ↑ SOD/CAT; ↓ lipid/protein oxidation; improved hepatic, renal, pancreatic and cardiac function	[63,97,98,99]
Selenium	Obesity and diet-induced hepatic injury models; high-fructose-fed mice	KEAP1/Nrf2; GPx3; selenoproteins; SEPP1; mitochondrial pathways	Selenoprotein-dependent antioxidant defense; activation of Nrf2; mitochondrial protection	↓ hepatic fat accumulation; improved insulin sensitivity/glucose tolerance; ↓ oxidative stress/inflammation; improved mitochondrial function	[100,101,102,103,105,161]
N-acetylcysteine (NAC)	HFD models; offspring of obese mothers; HFD male mice; HFD-exposed animals	GSH; TXNIP/NLRP3; IL-6/JAK2/STAT3/FOXO6; GPX4	Cysteine donation and GSH replenishment; attenuation of ROS-dependent inflammasome activation; inhibition of lipid ROS/ferroptosis	↓ oxidative stress; improved insulin sensitivity; ↓ hepatic steatosis/fibrosis; neuroprotection; improved cardiac redox homeostasis	[45,62,106,108]
Superoxide dismutase (SOD)/SOD mimetics	HFD-fed mice; obese Zucker rats; NAFLD models	SOD2/MnSOD; SOD3; mitochondrial superoxide	Conversion of superoxide to H_2_O_2_; reduction in superoxide burden; restoration of mitochondrial redox balance	↓ visceral adiposity; ↓ adipocyte death/inflammation; improved insulin sensitivity; ↓ oxidative injury	[31,40,64,78,79,111,112]
Glutathione (GSH)/GSH-related strategies	Dexamethasone-induced insulin resistance; diet-induced NAFLD; obese skeletal muscle	GSH/GSSG; GPx; SOD; Nrf2	Detoxification of peroxides; maintenance of protein thiols and cellular redox potential; preservation of GSH/GSSG balance	↓ MDA and oxidative damage; improved insulin sensitivity; improved muscle function; attenuation of inflammatory signaling	[63,114,162]
Coenzyme Q10 (CoQ10/ubiquinol)	HFD mice; palmitate-treated hepatocytes; KKAy obese/diabetic mice; HFD/high-fructose rats	AMPK; SIRT1/PGC-1α/PPARα; mitochondrial electron transport	Mitochondrial electron-carrier antioxidant activity; ↓ superoxide and lipid peroxidation; support of endogenous GSH/antioxidant systems	↓ hepatic oxidative stress; ↓ inflammation; improved insulin signaling; improved mitochondrial function; reduced adipose expansion	[36,116,117,163]
α-Lipoic acid (ALA)	Obese rodents; HFD-fed rats; visceral/subcutaneous adipose tissue; obese vascular models	AMPK; GSH; NOX; NF-κB; redox-sensitive signaling	Reduction in oxidative/nitrosative stress; enhancement of glutathione redox status; suppression of NOX/NF-κB signaling	↓ lipid peroxidation; ↓ adipose inflammation/apoptosis; improved β-oxidation; improved vascular function	[37,119,120,121]
L-Carnitine	High-fructose rats; hereditary hypertriglyceridemic rats; MCD-induced steatohepatitis; HFD-induced NASH	Nrf2/KEAP1; GPx; GSH/GSSG; NADPH oxidase; mitochondrial fatty acid transport	Improved mitochondrial fatty acid oxidation; activation of Nrf2; enhancement of endogenous antioxidant defenses; reduction in NOX-derived ROS	↓ hepatic steatosis; ↓ oxidative stress/fibrosis; improved insulin sensitivity; improved vascular and cardiac function	[35,123,124,125,126,164,165]
β-Alanine/Carnosine	HFD-fed rats; cardiometabolic stress models	Carnosine; 4-HNE; NF-κB/MAPK; carbonyl stress	Increases carnosine availability; scavenging of reactive carbonyls and 4-HNE; reduction in protein carbonylation	↓ lipid peroxidation; ↓ AGE/ALE formation; ↓ inflammation; improved insulin resistance-associated metabolic dysfunction	[91,113,127,128,129,130,143,151]
Taurine	HFD-fed rodents and obese ICR mice	SOD, GPx, CAT; PPARγ; C/EBPs; PGC-1α; UCP1; NF-κB	↑ endogenous antioxidant enzymes; ↓ ROS/MDA; mitochondrial protection; attenuation of redox-inflammatory signaling	↓ adipogenesis/lipogenesis; ↑ thermogenesis; ↓ macrophage infiltration; ↓ inflammatory cytokines; improved mitochondrial integrity	[131,132,133,134]
Melatonin	HFD-fed mice; Zucker diabetic fatty rats; obese mice	SOD/CAT; AMPK; FGF21; OPA1/DRP1; UCP1; PGC-1α; PRDM16	Direct/indirect antioxidant activity; mitochondrial stabilization; reduction in ROS and lipid peroxidation	↓ adipocyte hypertrophy; ↓ hepatic steatosis/inflammation; ↑ thermogenesis; improved mitochondrial dynamics; ↓ oxidative damage	[75,135,136,166]
Fenofibrate *	HFD mice; HFD-induced NAFLD; high-fat/high-fructose rats; HFD nephropathy	PPARα; UCP1; PGC-1α; PRDM16; AMPK; autophagy	Indirect redox effect through enhanced fatty acid oxidation, peroxisomal function and reduction in lipid overload	↓ adipocyte size; ↑ fatty acid oxidation/thermogenesis; ↓ hepatic oxidative damage; improved glucose/lipid metabolism; renal protection	[91,137,138,139,140]
Exenatide *	HFD-induced obese mice; diet-induced steatohepatitis; NAFLD; HFD renal models	GLP-1R/cAMP/PKA; PI3K/Akt; SIRT1/PGC-1α; PINK1/Parkin; NLRP3	Primarily metabolic/mitochondrial effects with secondary reduction in ROS; mitochondrial quality control and mitophagy	↓ hepatic/renal oxidative stress; improved insulin sensitivity; ↓ lipotoxicity; ↓ NLRP3 activation; improved mitochondrial function	[141,142,143]
Metformin *	HFD-fed obese mice; ob/ob mice; HFD nephropathy; obese insulin-resistant rats	AMPK; Nrf2/HO-1; mitochondrial complex I; SIRT1/PGC-1α; NOX2/NOX4	Secondary redox modulation through AMPK activation, mild complex I inhibition, Nrf2 activation and reduction in NOX-derived ROS	↓ oxidative stress; improved insulin sensitivity; ↓ steatosis/inflammation; mitochondrial, renal and vascular protection	[144,146,147,148,149,150]
Orlistat *	Obese rats; HFD-induced MAFLD/NASH; obese insulin-resistant rats	Dietary fat absorption; mitochondrial ROS; NOX; NF-κB; GSH	Indirect redox effect through reduced dietary fat absorption, circulating FFA and lipotoxic substrate availability	↓ body weight; ↓ lipid peroxidation; ↓ hepatic oxidative stress/inflammation; restored antioxidant defenses	[151,152,167]
Sibutramine *	Wistar rats treated for 28 days	Norepinephrine/serotonin reuptake; PI3K/Akt redox-sensitive signaling	Not an antioxidant strategy; preclinical evidence indicates reduced antioxidant capacity and increased lipid peroxidation	↓ SOD/CAT/GPx; ↓ GSH/urate; ↑ TBARS; potential pro-oxidant redox profile	[153,154,155,156]
Salicylic-acid nitroalkene (SANA)	HFD-fed mice	Electrophilic signaling; KEAP1/Nrf2; NF-κB; NOX; mitochondrial/creatine cycling	Potential adaptive redox signaling through electrophilic modification of cysteine residues and Nrf2 activation; mechanism remains incompletely defined	↓ body-weight gain; ↑ glucose tolerance; ↓ hepatic lipid accumulation; ↑ energy expenditure; ↓ lipid peroxidation/inflammation	[77,159]

The table summarizes the antioxidant and redox-modulating interventions investigated in experimental models of obesity, including vitamins, minerals, amino acids, endogenous antioxidant systems, natural compounds, and pharmacological agents. For each intervention, the main experimental models, molecular targets and pathways, proposed antioxidant or redox mechanisms, and major reported metabolic effects are presented. Downward arrows indicate a reduction in the observed effect, whereas upward arrows indicate an increase in the observed effect. Pharmacological agents marked with an asterisk (*) are not considered conventional antioxidant therapies; their effects on oxidative stress and redox homeostasis are primarily indirect or secondary to their principal pharmacological mechanisms.

## Data Availability

No new data were created or analyzed in this study. Data sharing is not applicable to this article.

## Abbreviations

The following abbreviations are used in this manuscript:

AGE	Advanced glycation end-products
BAT	Brown adipose tissue
BMI	Body Mass Index
BSEP	Bile salt export pump
CAT	Catalase
CBR1	Carbonyl reductase-1
CK-MB	Creatine kinase-MB
CPT1A	Carnitine palmitoyltransferase 1
CYP7A1	Cytochrome P450 family 7 subfamily A member 1
DNA	Deoxyribonucleic acid
DRP1	Dynamin-related protein 1
ERO1	Endoplasmic reticulum oxidoreductin 1
FAS	Fatty acid synthase
FGF21	Fibroblast growth factor 21
FOXO	Forkhead box O
FTO	Fat mass and obesity-associated gene
FXR	Farnesoid X receptor
GFR	Glomerular filtration rate
GLP-1R	Glucagon-like peptide-1 receptor
GPx	Glutathione peroxidase
GSH	Reduced glutathione
GSSG	Oxidized glutathione
HFD	High-fat diet
HFSD	High-fat, high-sucrose diet
HMGB1	High mobility group box 1
HOMA-IR	Homeostatic Model Assessment for Insulin Resistance
IFN	Interferon
IGF2	Insulin-like growth factor 2
IKK	IkappaB kinase
IL	Interleukin
JAK	Janus kinase
KEAP1	Kelch-like ECH-associated protein 1
LC	L-carnitine
LDH	Lactate dehydrogenase
LDL	Low-density lipoprotein
LF/HF	Low frequency/High frequency
LOOH	Lipid hydroperoxides
MAFLD	Metabolic-associated fatty liver disease
MAPK	Mitogen-activated protein kinase
MASLD	Metabolic dysfunction-associated steatotic liver disease
MC4R	Melanocortin 4 receptor
MDA	Malondialdehyde
NAC	N-acetylcysteine
NADH	Nicotinamide adenine dinucleotide
NADPH	Nicotinamide adenine dinucleotide phosphate
NAFLD	Non-alcoholic fatty liver disease
NASH	Non-alcoholic steatohepatitis
NBT	Nitroblue tetrazolium
NF-kB	Nuclear factor kappa B
NGAL	Neutrophil gelatinase-associated lipocalin
NLRP3	NLR family pyrin domain containing 3
NOX	NADPH oxidase
NRF2	Nuclear factor erythroid 2-related factor 2
OPA1	Optic atrophy 1
PGC	Peroxisome proliferator-activated receptor gamma coactivator
PI3K	Phosphoinositide 3-kinase
PINK1	PTEN-induced kinase 1
PKA	Protein kinase A
PKC	Protein kinase C
PLP	Pyridoxal phosphate
PPAR	Peroxisome proliferator-activated receptor
PRDM16	PR domain containing 16
PUFA	Polyunsaturated fatty acids
RA	Retinoic acid
RAGE	Receptor for advanced glycation end products
RAR	Retinoic acid receptor
RNS	Reactive nitrogen species
ROS	Reactive oxygen species
RVLM	Rostral ventrolateral medulla
SANA	Salicylic-Acid Nitroalkene
SCOUT	Sibutramine Cardiovascular Outcomes Trial
SEPP1	Selenoprotein P
SIRT1	Sirtuin 1
SOD	Superoxide dismutase
STAT3	Signal transducer and activator of transcription 3
TBARS	Thiobarbituric acid reactive substances
TLR4	Toll-like receptor 4
TNF	Tumor necrosis factor
TXNIP	Thioredoxin-interacting protein
UCP1	Uncoupling protein 1
WAT	White adipose tissue
WHO	World Health Organization
ZDF	Zucker diabetic fatty
ZO-1	Zonula occludens-1
